# Reduction of Human Glioblastoma Spheroids Using Cold Atmospheric Plasma: The Combined Effect of Short- and Long-Lived Reactive Species

**DOI:** 10.3390/cancers10110394

**Published:** 2018-10-23

**Authors:** Angela Privat-Maldonado, Yury Gorbanev, Sylvia Dewilde, Evelien Smits, Annemie Bogaerts

**Affiliations:** 1 PLASMANT, Chemistry Department, University of Antwerp, 2610 Antwerp, Belgium; yury.gorbanev@uantwerpen.be (Y.G.); annemie.bogaerts@uantwerpen.be (A.B.); 2 Solid Tumor Immunology Group, Center for Oncological Research, University of Antwerp, 2610 Antwerp, Belgium; evelien.smits@uantwerpen.be; 3 Protein Chemistry, Proteomics and Epigenetic Signaling, Department of Biomedical Sciences, University of Antwerp, 2610 Antwerp, Belgium; sylvia.dewilde@uantwerpen.be

**Keywords:** cancer, cold atmospheric plasma (CAP), spheroid shrinkage, cytotoxicity, tumour reduction, glioblastoma, short-lived reactive species, cell migration, proliferation

## Abstract

Cold atmospheric plasma (CAP) is a promising technology against multiple types of cancer. However, the current findings on the effect of CAP on two-dimensional glioblastoma cultures do not consider the role of the tumour microenvironment. The aim of this study was to determine the ability of CAP to reduce and control glioblastoma spheroid tumours in vitro. Three-dimensional glioblastoma spheroid tumours (U87-Red, U251-Red) were consecutively treated directly and indirectly with a CAP using dry He, He + 5% H_2_O or He + 20% H_2_O. The cytotoxicity and spheroid shrinkage were monitored using live imaging. The reactive oxygen and nitrogen species produced in phosphate buffered saline (PBS) were measured by electron paramagnetic resonance (EPR) and colourimetry. Cell migration was also assessed. Our results demonstrate that consecutive CAP treatments (He + 20% H_2_O) substantially shrank U87-Red spheroids and to a lesser degree, U251-Red spheroids. The cytotoxic effect was due to the short- and long-lived species delivered by CAP: they inhibited spheroid growth, reduced cell migration and decreased proliferation in CAP-treated spheroids. Direct treatments were more effective than indirect treatments, suggesting the importance of CAP-generated, short-lived species for the growth inhibition and cell cytotoxicity of solid glioblastoma tumours. We concluded that CAP treatment can effectively reduce glioblastoma tumour size and restrict cell migration, thus demonstrating the potential of CAP therapies for glioblastoma.

## 1. Introduction

Glioblastoma multiforme (GBM) is a highly aggressive neoplasia and the most common aggressive tumour on the central nervous system in the adult population [1]. Current therapies against GBM include maximal surgical resection, radiation and chemotherapy. However, tumour recurrence is highly common, which contributes to the poor survival rates after diagnosis (median survival of 15 months; five-year survival of less than 5%) [2,3]. Despite significant improvements in cancer treatment, GBM remains an incurable neoplasia.

Cold atmospheric plasmas (CAPs) are currently being explored due to their anti-cancer properties [4,5]. The chemical components produced by CAPs, identified as the active agents responsible for the biological effects, have been extensively studied [6,7]. The reactive oxygen and nitrogen species (RONS) generated and delivered by plasma cause functional and structural damage to lipids in cell membranes [8], oxidize proteins [9], induce DNA breaks [10], and promote cell death by different mechanisms [11,12]. In contrast, the physical components produced by plasma such as UV photons and electromagnetic fields on their own have a negligible cellular impact [13]. Furthermore, it has been suggested that CAP is selective towards cancer cells due to the increased presence of aquaporins and the reduced amount of cholesterol in their cell membranes [14,15], which facilitates the access of RONS into the cell. Cancer cells are also less effective in removing extracellular H_2_O_2_ compared to normal cells [16] (one of the main reactive species produced by CAP), adding to the selectivity of CAP treatments.

The cytotoxic effect of CAPs in glioblastoma has been mainly demonstrated using the conventional two-dimensional (2D) monolayer cell model. Previous studies on glioblastoma cells have shown that CAP reduces the cell viability and induces apoptosis [17]. Even more, CAP treatment reduces clonogenicity and induces cell-cycle arrest in glioblastoma cells resistant to temozolomide, a chemotherapeutic agent used to treat glioblastoma patients [18]. The biomedical effects of CAPs are achieved not only by direct treatment, but also by applying plasma-treated liquids, rich in long-lived species [19,20]. These studies carried out using the 2D monolayer model, although informative, do not reproduce the biological microenvironment of tumours. Recent findings indicate that the tumour microenvironment plays a key role in the response to treatment, regulating tumour progression and metastatic processes [21]. Thus, it is possible that the therapies developed using 2D systems would not meet the requirements to achieve the desired response in three-dimensional (3D) tumours. To date, only few studies have used more complex models such as in vitro 3D culture systems to assess the effect of CAP on different types of cancer [22,23,24] and one study has tested CAP on glioblastoma tumours in mice [25].

The COST plasma jet was developed as a standard reference plasma jet [26,27]. Our group has previously reported the anticancer capacity of this CAP on 2D glioblastoma cultures [17]. In the present study, we used an in vitro 3D tumour spheroid model that allows cells to behave in a manner that is closer to natural conditions. The model is more relevant than 2D cultures as it enables the in vitro spheroid to develop biophysical properties characteristic of solid tumours (nutrients and oxygen gradients, catabolite accumulation) and the synthesis and assembly of extracellular matrix (ECM) proteins. In this environment, the cells in the spheroid conformation also present proliferation gradients and gene expression profiles closer to the clinical and in vivo models [28,29]. Using this model, we investigated the ability of CAP to reduce tumour size in glioblastoma spheroids in vitro. Both biological and chemical assays were performed to assess the effect of CAP treatments in U87-Red and U251-Red glioblastoma spheroids. We found that both short- and long-lived species were required to exert an efficient inhibitory effect. Our approach thus highlights the need of plasma-generated short-lived RONS to reduce tumour size and control tumour growth, and emphasizes the importance of the tumour microenvironment for the development of more effective plasma therapies.

## 2. Results

### 2.1. Identifying an Effective Plasma Treatment

The purpose of our study was to determine the effect of plasma treatment on a 3D tumour spheroid in vitro. We used red-fluorescent cells to facilitate the identification of living cells. The spheroid core (red, proliferative and viable part of the spheroid) was monitored to determine variations in its area relating to growth inhibition or spheroid shrinkage upon CAP treatment. The total spheroid area was measured to assess changes in spheroid size due to cell death and destruction of the spheroid architecture. Cell death in glioblastoma spheroids was monitored using the Cytotox Green reagent, a DNA-binding dye that produces a fluorescent signal in cells with a damaged cell membrane.

We first assessed the cytotoxic effect of a single direct plasma treatment on glioblastoma spheroids in phosphate buffered saline (PBS) using the COST jet with dry He, He + 5% H_2_O or He + 20% H_2_O. No cytotoxic effect or inhibition of spheroid growth was achieved with the plasma generated using dry He or He + 5% H_2_O (not shown). However, we observed a delay in spheroid growth in U87-Red and U251-Red, as well as cell death in U87-Red spheroids treated with the COST jet He + 20% H_2_O (Supplementary Information, Figure S1). Admittedly, this effect was not enough to inhibit spheroid growth or to decrease the spheroid core area. Thus, we administered an additional plasma treatment at 24 h.

**Figure 1:**
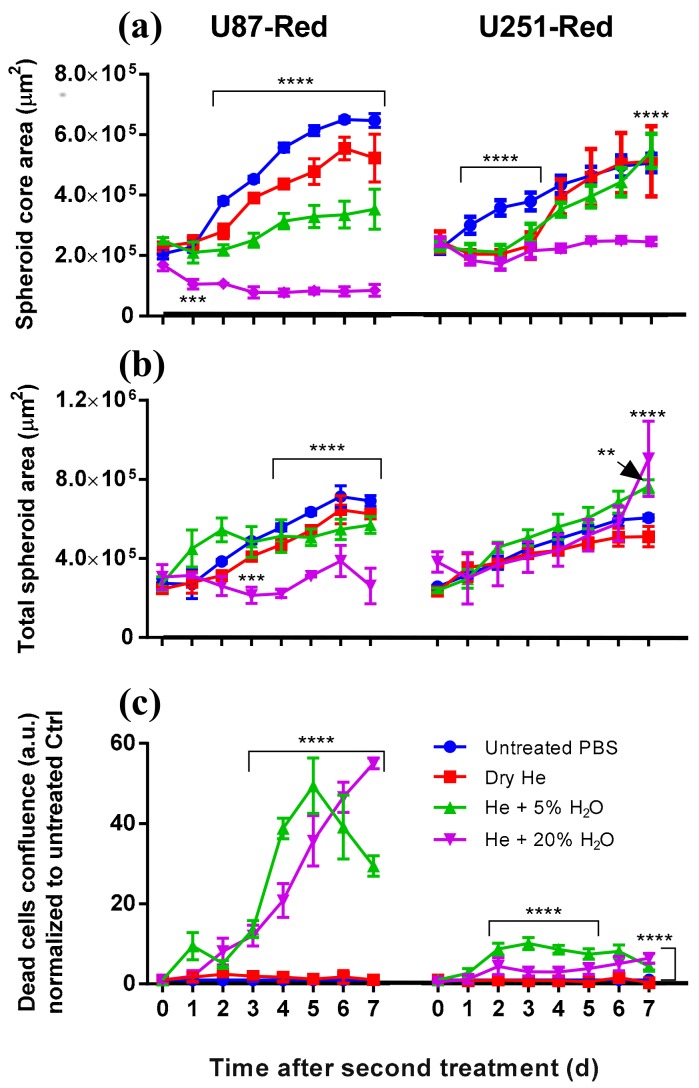
Consecutive plasma treatment inhibited growth in glioblastoma spheroids. (**a**) The area of the spheroid core (viable cells) was reduced by 2× direct plasma treatment generated with dry He, He + 5% H_2_O or He + 20% H_2_O vapour saturation. The most effective inhibition of growth in both U87-Red and U251-Red was achieved using plasma generated with He + 20% H_2_O (*p* ≤ 0.0001). (**b**) Similar values of the total spheroid area (comprising living spheroid core and dead cells) were obtained for all the conditions in both cell lines, except for U87-Red, He + 20% H_2_O, where the total spheroid area was smaller. (**c**) The addition of H_2_O to the gas feed resulted in increased cytotoxicity for both cell lines. Cell death is expressed as the ratio of Cytotox Green^+^ cells in treated spheroids/untreated controls at each time point (a.u.). Dead cells confluence = confluence percentage of the image area occupied by dead cells. Results corresponding to U87-Red (**left**) and U251-Red spheroids (**right**). Data representative of two independent experiments, 4–6 spheroids per condition. Mean ± SD; ** = *p* ≤ 0.01; **** = *p* ≤ 0.0001.

**Figure 2:**
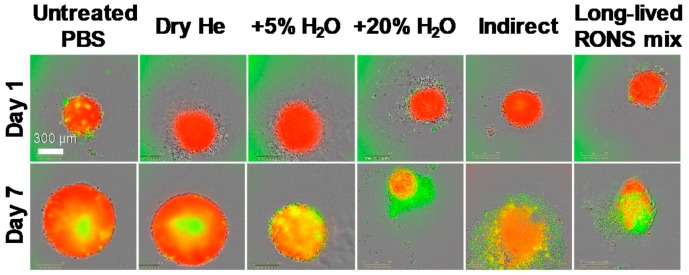
Spheroid growth/shrinkage upon the different treatments applied. Representative images of spheroid growth/shrinkage and cytotoxic effect in U87-Red spheroids on day 1 and 7 days after exposure to the different treatments tested. Living cells in red, Cytotox Green^+^ cells in green. Scale bar = 300 µm.

The effective outcome was achieved after the 2× direct treatment by He + 20% H_2_O plasma. Under this condition, the plasma not only inhibited spheroid growth, but also reduced the spheroid core area. This effect was maintained seven days post-treatment in U87-Red spheroids (*p* ≤ 0.0001; Figure 1a and Figure 2). In contrast, spheroids 2×-treated with He + 5%H_2_O and dry He plasma reduced their spheroid core area, but the spheroid growth resumed after 72 h in both cell lines (Figure 1a). He + 5%H_2_O and dry He plasma-treated U251-Red spheroids yielded the same core area as untreated controls (*p* ≤ 0.0001; Figure 1a), evidencing a lower response to plasma treatment. The analysis of the total spheroid area evidenced similar values for all the conditions tested in both cell lines, except for He + 20% H_2_O in U87-Red (Figure 1b). CAP treatment was cytotoxic only when H_2_O was added to the feed gas (*p* ≤ 0.0001; Figure 1c), which suggests the participation of reactive oxygen species (ROS) in the overall biological effect. The least effective treatment was with dry He, where only a reduction in spheroid growth but no cytotoxic effect was observed (similar levels to untreated PBS control, Figure 1). This could suggest a modulatory rather than a cytotoxic effect of dry He plasma on glioblastoma cells. We concluded that the 2× He + 20% H_2_O plasma treatment effectively inhibited spheroid growth and had a cytotoxic effect in U87-Red and U251-Red glioblastoma spheroids.

### 2.2. RONS Present in Plasma-Treated PBS (pPBS)

**Figure 3:**
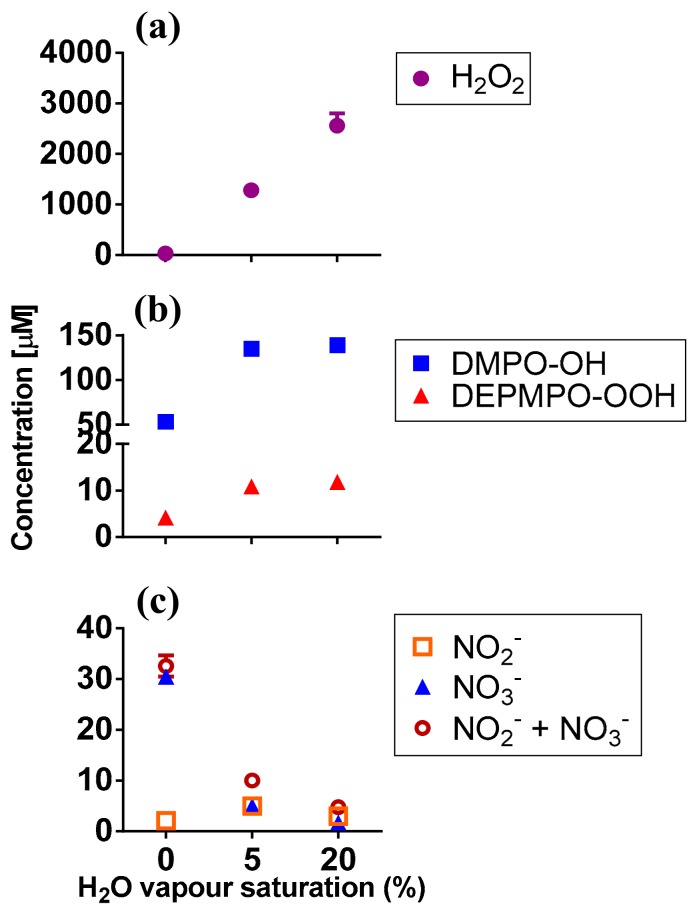
Short- and long-lived RONS present in plasma-treated PBS. (**a**) H_2_O_2_; (**b**) DMPO-OH and DEPMPO-OOH adducts; (**c**) NO_2_^−^, NO_3_^−^ and NO_2_^−^ + NO_3_^−^ were detected in plasma-treated PBS by EPR and colourimetry (see Mat. and Met.). Experiments were carried out in 200 μL pPBS, using dry He, He + 5% or 20% H_2_O vapour saturation. Data represent mean values (*n* ≥ 2).

To determine which RONS could be responsible for the biological outcome described above, we measured the short- and long-lived species present in pPBS by electron paramagnetic resonance (EPR) and colourimetry. H_2_O_2_ was found in pPBS in high concentrations. The concentration was dependent on the H_2_O vapour saturation of the feed gas: a higher percent of H_2_O saturation resulted in more H_2_O_2_ production (Figure 3a), in agreement with our previous report for this plasma jet [27]. The short-lived species cannot be detected directly and requires the use of spin traps (which react with radicals to form more persistent nitroxide radical adducts) [30]. We used 5,5-dimethyl-1-pyrroline *N*-oxide (DMPO) to detect •OH, and 5-(diethoxyphosphoryl)-5-methyl-1-pyrroline *N*-oxide (DEPMPO) for O_2_•^–^/•OOH radicals (Figures S2 and S3) [30,31,32]. DMPO-OH and DEPMPO-OOH adducts were detected in the μM range. Their concentration increased with the increasing H_2_O vapour content of the feed gas (Figure 3b), again in agreement with the previously reported data [27].

We note that the concentration of the spin adducts did not correspond to the total amount of the radical induced by CAP in PBS. However, the changes in their concentration under different plasma conditions corresponded to the changes in the radical amount [31,33]. Additionally, the nitroxide moiety of spin adducts was prone to decay in plasma-treated liquids [34]. The direct comparison of different conditions is possible if the concentration of a spin adduct does not decay or reach a flat plateau within the experimental (plasma exposure) time frame [33,35]. We demonstrate here that the DMPO-OH concentration increased near-linearly within the experimental time frame (up to 4 min), thus confirming the applicability of the method (Figure S4).

The spin trap 2,2,6,6-tetramethylpiperidine (TEMP) was used to detect the oxygen species produced by plasma, such as O, O_2_(a^1^Δg), and O_3_ [31,33]. The use of NaN_3_, an O_2_(a^1^Δg) scavenger, allowed to determine the specific contribution of O_2_(a^1^Δg) to the formation of 2,2,6,6-tetramethylpiperidine 1-oxyl (TEMPO), a stable and EPR-detectable nitroxide radical, via the oxidation of TEMP. However, we only detected minor amounts of the formed TEMPO (below 9 µM) compared to the impurities in untreated samples (Figure S5), and they did not change with varied H_2_O saturation of the feed gas or introduction of NaN_3_ (not shown). Previously, we have demonstrated a very efficient plasma-induced degradation of TEMPO derivatives [34]. Hence, we attribute the observed effect to two factors: (1) low concentrations of O/O_2_(a^1^Δg)/O_3_ produced by plasma in the presence of water vapour [31]; and (2) rapid degradation of the formed TEMPO by •OH radicals which are present in high concentrations (see Figure 3b).

We also attempted to detect •NO radicals using N-methyl-d-glucamine dithiocarbamate (MGD)-iron(II) complex ((MGD)_2_Fe^2+^) and 2-phenyl-4,4,5,5-tetramethylimidazoline 1-oxyl 3-oxide (PTIO) spin traps, as described in Section 4.8 (see Mat. and Met.). Under all plasma conditions, no •NO was detected. These results agreed with the low concentrations of other measured (long-lived) reactive nitrogen species (RNS) induced by CAP in pPBS (Figure 3c).

Thus, we can tentatively ascribe the biological effects of CAP to the following species: •OH, O_2_•^−^/•OOH, H_2_O_2_, NO_2_^−^/NO_3_^−^. We acknowledge that other biologically-relevant, plasma-induced species such as ClO^−^ [36], ONOO^−^ [37,38] and OONOO^−^ [39] could be present in pPBS, but these were not monitored in this study. However, the two latter species are likely to be present in low concentrations (if any), like all other detected RNS, and ClO^−^ is formed in large quantities, mostly in the presence of oxygen species such as atomic oxygen [36].

### 2.3. Determining the Role of the Short- and Long-Lived Species in Plasma on Spheroid Viability

To determine whether the biological effect observed was due only to the long-lived species or to the combination of short- and long-lived species produced by CAP, we assessed the effect of: (1) 2× direct plasma treatment; (2) 2× indirect plasma treatment (2× pPBS); and (3) 2× RONS mix in PBS (with the same concentrations of the long-lived species H_2_O_2_ and NO_2_^−^ as in the pPBS used) on glioblastoma spheroids. NO_2_^−^ can undergo further oxidation in the liquid to yield NO_3_^−^ [40]. Thus, we used NO_2_^−^ in the concentration that corresponded to the combined amount of NO_2_^−^ and NO_3_^−^. We acknowledge that the actual concentration of NO_2_^−^ induced in PBS by CAP may have been lower.

**Figure 4:**
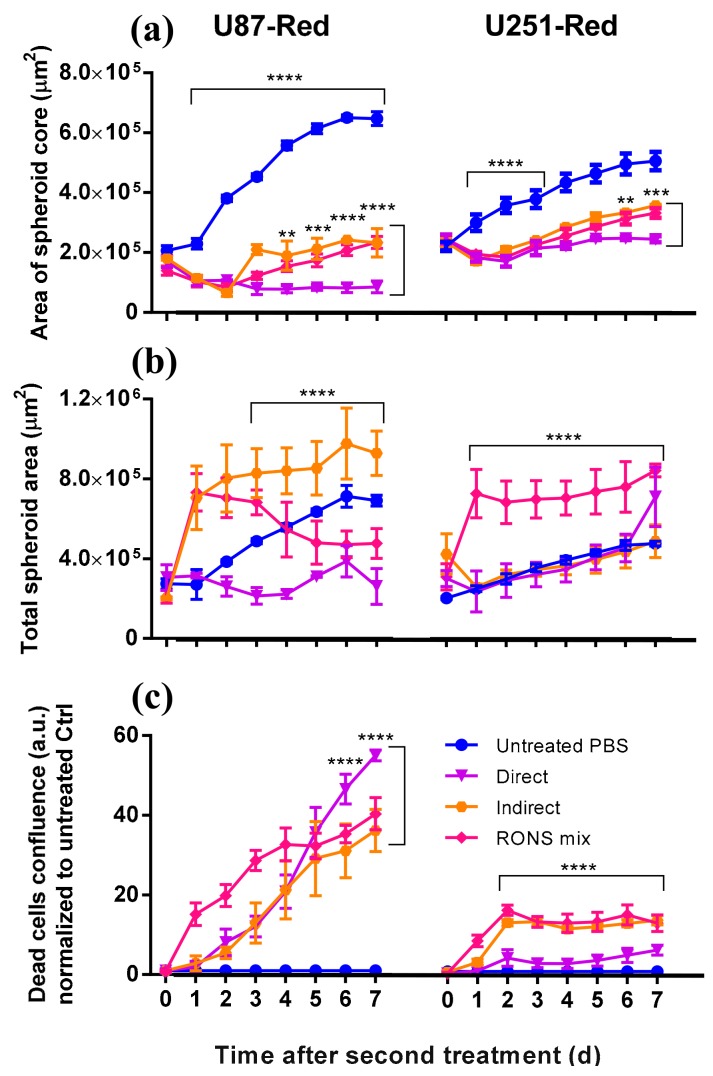
Short- and long-lived species delivered by plasma are needed to inhibit spheroid growth. (**a**) A statistically significant difference was observed in the area of the spheroid core (viable cells) in spheroids exposed to 2× direct plasma treatment compared to those treated with pPBS (indirect) and the RONS mix (U87-Red, *p* ≤ 0.0001; U251-Red, *p* ≤ 0.001). (**b**) U87-Red spheroids treated with pPBS and the RONS mix showed higher total spheroid area than spheroids treated 2× directly with plasma (*p* ≤ 0.0001). In U251-Red spheroids, higher total spheroid area was observed only in spheroids treated with the RONS mix. (**c**) The amount of cell death induced in spheroids by pPBS and RONS mix were comparable. Cell death is expressed as the ratio of Cytotox Green^+^ cells in treated spheroids/untreated controls at each time point (a.u.). Dead cells confluence = confluence percentage of the image area occupied by dead cells. Results corresponding to U87-Red (**left**) and U251-Red (**right**) spheroids. Data representative of two independent experiments, 4–6 spheroids per condition. Mean ± SD; ** = *p* ≤ 0.01; *** = *p* ≤ 0.001; **** = *p* ≤ 0.0001.

The 2× treatments with either pPBS or RONS mix were less effective than the 2× direct treatment in inhibiting spheroid growth in both glioblastoma cell lines, especially in U87-Red (*p* ≤ 0.0001; Figure 2 and Figure 4a). The direct treatment induced spheroid shrinkage in U87-Red that was maintained for the whole duration of the experiment, whereas spheroid growth resumed 72 h after in the spheroids exposed to 2× pPBS and 2× RONS mix. Although the 2× direct plasma treatment was not able to completely inhibit U251-Red spheroid growth (Figure 4a), a statistically significant difference was observed between this treatment and the other two conditions tested here (*p* ≤ 0.001 at t = 168 h). Thus, we conclude that the 2× direct plasma treatment was more efficient than the 2× pPBS and RONS mix in inhibiting spheroid growth.

In both cell lines, spheroids exposed to 2× RONS mix showed an increased total spheroid area compared to the untreated control (Figure 4b). In U87-Red, this was also observed in 2× pPBS-treated spheroids. This effect could be explained by the cell detachment of dead cells from the spheroid core that add to the total spheroid area (Figure 4c). However, we observed that the high cytotoxicity of the 2× direct treatment (Figure 4c) did not correlate with an increase in the total spheroid area (Figure 4b). Thus, it appears that the 2× direct treatment was able not only to inhibit spheroid growth and kill cancer cells as shown here, but also to restrict the cell detachment of dead cells and spheroid tissue from the spheroid core. Although we acknowledge the participation of the long-lived species present in pPBS and in the RONS mix, our results indicate that the short-lived species delivered by plasma during the 2× direct treatment are required to fully inhibit spheroid growth in U87-Red and to delay it in U251-Red.

### 2.4. The 2× Direct Plasma Treatments Inhibited Cell Migration

**Figure 5:**
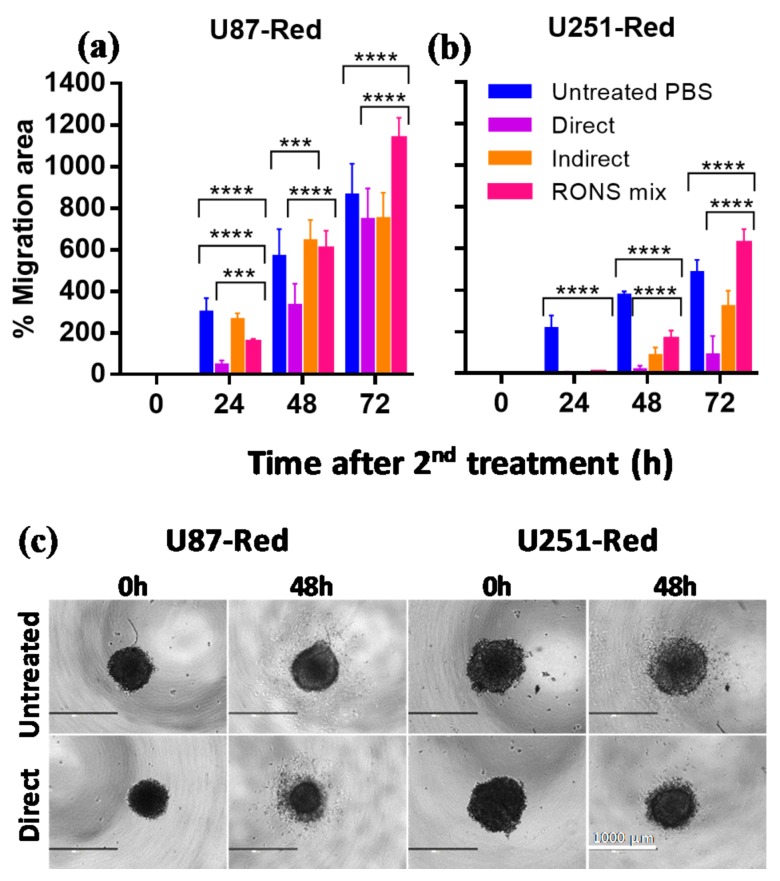
The migratory ability of U87-Red and U251-Red cells is affected by the 2× direct plasma treatment. The 2× direct plasma treatment inhibited cell migration for (**a**) up to 48 h in U87-Red and (**b**) up to 72 h in U251-Red. 2× pPBS and RONS mix had a significant but less inhibitory effect, reaching similar migration areas than the untreated PBS controls after 48 h (U87-Red) and 72 h (U251-Red). (**c**) Representative images of U87-Red and U251-Red cell migration (0 and 48 h after consecutive treatments). % Migration area = 100*(area T_x_-T_0_/area T_0_). Data representative of two independent experiments, 4–6 spheroids per condition. Mean ± SD; *** = *p* ≤ 0.001; **** = *p* ≤ 0.0001. Scale bar = 1000 µm.

Glioblastomas are characterized by their high migratory nature, which allows them to metastasize into surrounding brain tissues [1]. We investigated the ability of our highly cytotoxic direct plasma treatment (2× plasma treatment, He + 20% H_2_O) to inhibit the migration of the cells that survive the treatment. Our results demonstrated that the 2× direct plasma treatment reduced cell migration (Figure 5a,b). The 2× direct treatment had a significant effect on both cell lines, with little or no cell migration 24 h post-treatment (*p* ≤ 0.0001) and a hindered migration in U87-Red cells 48 h post-treatment (*p* ≤ 0.0001; Figure 5a,b). This effect was maintained 72 h post-treatment only in U251-Red spheroids (*p* ≤ 0.0001). Lower cell migration inhibition was observed in 2× pPBS- and RONS mix-treated spheroids at all time points (except after 48 h for U87-Red and 72 h for both cell lines). We observed morphological changes in the spheroids upon plasma treatment, specifically the shrinkage of the spheroid core and presence of dead cells around it (Figure 5c). Whereas control spheroids presented a homogeneous migration pattern with cells spreading in all directions around the spheroid core, the pattern was asymmetrical in plasma-treated spheroids with a reduced number of migratory cells. This could be due to the amount of dead cells in the outer layer of the spheroid. The differences observed between the migration dynamics of both cell lines could also indicate different survival strategies.

Based on these results, we concluded that the 2× direct plasma treatment effectively inhibited the migratory ability of U87-Red and U252-Red spheroids and this treatment was more efficient than the pPBS and RONS mix. Thus, the combination of short-lived species delivered by CAP is needed to effectively reduce cell migration in glioblastoma spheroids.

### 2.5. ki67 Expression Was Reduced by 2× Direct Plasma Treatment

**Figure 6:**
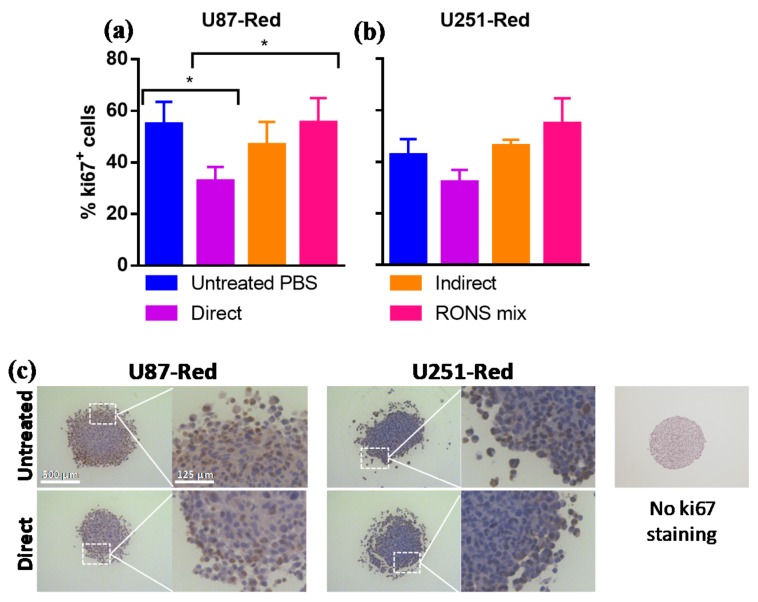
The 2× direct plasma treatments reduced the expression of the proliferative marker ki67 in glioblastoma spheroids. The 2× direct plasma treatment decreased the % ki67^+^ cells in (**a**) U87-Red spheroids (*p* < 0.05) and was lower than in those untreated or treated with pPBS and RONS mix (*p* < 0.05). (**b**) The same trend was observed in U251-Red spheroids (*p* > 0.05). The staining was scored using IHC Profiler in ImageJ. (**c**) Representative images of ki67 staining of U87-Red and U251-Red spheroids exposed to untreated PBS or He + 20% H_2_O direct treatment. Enlarged image demonstrates variable ki67 staining. No ki67 staining control on the right. Data representative of two independent experiments, 4–6 spheroids per condition. Mean ± SD; * = *p* < 0.05. Scale bar = 500 µm.

To further characterize the inhibitory ability of our plasma treatment, the expression of the proliferation marker ki67 in spheroids was assessed by immunohistochemistry. The percentage of ki67^+^ cells was reduced in U87-Red spheroids exposed to the 2× direct plasma treatment (*p* < 0.05; Figure 6a). Although no statistically significant difference was found between untreated and 2× directly-treated U251-Red, a trend to a reduced ki67 expression in 2× direct plasma-treated cells was observed (Figure 6b). The pPBS and RONS mix treatments produced similar %ki67^+^ cells comparable to the untreated controls in both cell lines. In addition to the reduced ki67 expression, the CAP-treated spheroids presented a more fragile structure, as shown in Figure 6c. Altogether, our results suggest that our 2× direct plasma treatment reduced ki67 expression in glioblastoma spheroids. This reduction correlates with the inhibition of growth observed in the 2× plasma-treated spheroids, as described in Section 2.1 and Section 2.3.

## 3. Discussion

Although significant preclinical work has been done in the field of medical plasmas for cancer, there are still unknowns regarding the efficacy of these treatments in natural conditions. A major concern is the use of models that do not consider the tumour microenvironment, such as the widely used 2D cell culture model. This comes at the cost of developing therapies tailored for a 2D model that might not be able to control tumour cell growth in natural conditions due to physiological and structural differences [41]. To address this problem, plasma treatment in 3D glioblastoma spheroids was explored in this study for the first time. The model resembled the nutrient and oxygen gradients and architecture of solid tumours and provided a more accurate representation of treatment outcomes under 3D conditions.

In this study, we demonstrate the importance of short-lived species delivered by CAP for the reduction of 3D glioblastoma spheroids. Interestingly, a single plasma treatment that would induce cell cytotoxicity in the 2D culture model was not sufficient to inhibit spheroid growth or to induce spheroid shrinkage, thus demonstrating the importance of using 3D culture models. However, the 2× direct plasma treatment administered 24 h after the initial treatment generated with 20% vapour saturation elicited a long-lasting cytotoxic and inhibitory effect in U87-Red spheroids and in U251-Red spheroids, though to a lesser degree. Whereas the total spheroid area and spheroid core area of untreated spheroids were of similar magnitude (no cell death, no spheroid damage), the increased total spheroid area of plasma-treated spheroids could be due to other factors. It is possible that the combination of cell death and oxidation of ECM caused by RONS damaged the spheroid architecture, resulting in an increased total spheroid area. In turn, the high cytotoxic effect could at least partially be explained by the high concentration of H_2_O_2_ produced in the PBS and its diffusion to the intracellular compartment aided by aquaporins [42]. However, we observed that only the 2× direct plasma treatment induced complete growth inhibition in U87-Red spheroids and reduced spheroid growth in U251-Red, while 2× pPBS and RONS mix treatments failed to induce such effects. We tentatively attribute this difference to the short-lived reactive species, most likely ROS such as •OH and O_2_•^–^ radicals (as RNS were only formed in very low concentrations), present only during the direct plasma treatment. Both the cytotoxic effect and the concentration of ROS in our plasma treatment increased with the increase of H_2_O vapour content in the feed gas, confirming this hypothesis.

As cell migration is an important feature of malignant, metastatic cancers, we assessed the ability of glioblastoma spheroids to migrate upon plasma treatment. Cells that survived the 2× direct plasma treatment presented reduced migratory activity compared to those exposed to the pPBS and RONS mix. This is in agreement with the findings on human metastatic breast cancer cells, where plasma treatment significantly inhibited migration and invasion of BrCa cells and fibroblasts [43,44]. Besides oxidation, plasma can also reduce the expression of integrin, an important cell surface protein that participates in cell motility and adhesion [43]. This effect is independent of the cell cycle phase, as plasma affects cells in all stages of the cell cycle [45]. Thus, this effect could be due to the destruction of ECM components and the oxidation of cell adhesion molecules on the cell surface [12]. Further studies on the expression of adhesion molecules and ECM oxidation in glioblastoma spheroids upon CAP treatment are needed to better understand the effect of plasma on cell migration.

The brain tissue is particularly susceptible to oxidative stress due to its high requirement for oxygen and its poor defence mechanisms against oxidative stress [46]. The difference in the response to treatment observed between both glioblastoma cell lines studied here could be due to different sensitivities to oxidative stress as a consequence of specific mutations [17], to different proliferative rates or metabolic activity [47]. For example, p53 (p53) and p16 (encoded by CDKN2A) are tumour suppressor proteins that control the cell cycle and are associated with cancer development [48]. It has been reported that mutations in p53 and CDKN2A impair their corresponding protein function, favouring the accumulation of DNA damage and making cells more sensitive to oxidative damage [49,50]. We have previously confirmed that the p53 status of both cell lines remained the same after cell passage (data not shown). U87-Red (p53 wild type, CDKN2A*^mut^*) was more sensitive to plasma treatment than U251-Red (p53*^mut^*, CDKN2A*^mut^)*. Although p53*^mut^* cells are reported to be more sensitive to plasma [51], this was not observed in our study. Thus, the mechanism of action of plasma might be independent of p53 [52]. Mutations in other genes such as CDKN2A could contribute to the sensitivity to plasma [17]. The combination of multiple mutations present in cancer cell lines could provide one explanation for the different responses to CAP treatment obtained here. In addition, the difference in protein expression between both cell lines could also explain the differences in the treatment outcome, as it has been demonstrated that the U87 and U251 cell lines differ in their proliferation, migration, and invasion abilities [53].

Supporting the growth inhibition exhibited in the 2× direct plasma-treated spheroids, we found a reduction in the expression of the proliferative marker ki67 in U87-Red and to a lesser extent in U251-Red. This marker is present in cells in G_1_, S, G_2_ and mitosis and absent in quiescent cells [54]. We demonstrated that the 2× direct plasma treatment, unlike pPBS or RONS mix, decreased the percentage of ki67^+^ cells in spheroids of both cell lines. Ki67^+^ cells were mainly located in the outer layer of the spheroids, corresponding to the proliferative fraction of the tumour. Our results correlated with previous findings reporting a decreased ki67 expression in the pancreatic tumours of mice treated with plasma-treated medium [55] and in 3D colorectal spheroids directly exposed to a plasma jet [24]. It is worth noticing that a reduction in ki67 expression was observed in spheroids immediately after the application of the second CAP treatment. Due to technical reasons, it was not possible to collect the spheroids for immunohistochemistry at later stages without disturbing their overall integrity, as plasma reduced the spheroid size and affected the structure of the spheroid (as observed in Figure 2 and Figure 6c). We speculate that ki67 expression in CAP-treated spheroids could further decrease over time, correlating with the sustained inhibition of growth observed here. The anti-proliferative effect of plasma, combined with the cytotoxic effect displayed, contributed to the reduction and control of the glioblastoma spheroids described here. Thus, we concluded that the 2× direct plasma treatment, rich in short- and long-lived species, can decrease ki67 expression and reduce the proliferative ability of cancer cells.

The results obtained in the present work could explain the findings of Chen et al. in athymic nude mice [25]. In that study, CAP treatment reduced the size of glioblastoma tumours in immunocompromised mice. This could have been achieved by the combination of effects described in our work: damage to the tumour microenvironment, the induction of cell death, the reduction of cell proliferation and the inhibition of cell migration. Our findings correlate with the results obtained by Chen et al., as the direct CAP treatments effectively reduced the glioblastoma spheroid size. In addition, the findings support the need of CAP-generated short-lived species for more effective therapies against glioblastoma. Our findings support the benefits of using the 3D spheroid model for CAP research, as spheroids can provide more biological information on CAP treatment in a 3D structures, which are closer to natural conditions. The use of this technology reduces animal testing and allows high throughput analyses, bridging the gap between in vitro and in vivo models for anticancer CAP treatments.

To date, the 3D spheroid model has been used only in three studies to investigate the effect of plasma treatments on HTC116 colorectal and head and neck FaDu cancer cells [22,23]. While the most recent article indicates that four consecutive treatments with plasma-activated medium were needed to inhibit FaDu spheroid growth [23], our therapy was able to achieve tumour reduction with only two applications, as it does not rely solely on the presence of long-lived species such as H_2_O_2_ for its efficacy. In principle, liquid solutions with CAP-induced long-lived species may not require plasma for their generation: we were able to generate a RONS mix with commercially available chemicals. However, plasma is required to efficiently deliver short-lived species, which substantially contribute to the effects of CAP, as we demonstrate here.

This is the first report of plasma inducing sustained tumour shrinkage and growth inhibition in glioblastoma spheroids. Also for the first time, we demonstrated the direct correlation between the in-liquid identified plasma RONS and the biological effect of CAP on 3D spheroid models. We showed that the 2× plasma treatment with the COST jet caused the inhibition of both spheroid growth and cell migration, and ultimately a reduction in ki67 expression in glioblastoma tumour spheroids. Our results demonstrated the importance of short-lived species delivered by plasma in the overall inhibitory effect, as both contribute to the elimination of 3D glioblastoma tumours. Furthermore, the different responses to treatment observed between the cell lines studied here emphasize the need to use relevant models to investigate the response to treatment under more representative conditions. Our findings regarding glioblastoma spheroids support the potential of CAP as a tool against cancer. Further studies on 3D spheroids or more complex 3D in vitro models using co-cultures of glioblastoma cells and astrocytes could bring light to the effect of CAP on the ECM and the safety of CAP application on benign cells. Likewise, the CAP treatments described here could be applied to spheroids generated from primary cells or complex organoids that more closely resemble the architecture of solid tumours, as this is an indispensable step towards the development of more effective CAP therapies.

## 4. Materials and Methods

### 4.1. Cell Lines and Reagents

Human GBM cell lines U87 and U251 were obtained from the Cell Line Service GmbH and kindly provided by Dr. Nicolas Goffart (Université de Liège, ULG), respectively. Cells were grown in Dulbecco’s Modified Eagle’s medium (DMEM) supplemented with 10% foetal bovine serum (FBS, Gibco, Fisher Scientific, Merelbeke, Belgium) with 2 mM L-Glutamine (Life Technologies, Eggenstein, Germany) and maintained in a tissue culture incubator at 37 °C with 5% CO_2_. Cells were transduced with the Nuclight Red Lentivirus reagent (Essen Biosciences, Ann Arbor, MI, USA) using their standard transduction protocol. The transduced cells referred here to as U87-Red and U251-Red, expressed nuclear mKate2 red fluorescent protein.

### 4.2. Generation of Spheroids

Cell suspensions were prepared at 5 × 10^4^ cells/mL density in culture medium supplemented with 0.24% methylcellulose to enhance spheroid formation. Cells were seeded in ultra-low attachment (ULA) 96-well plates (round bottom, Corning Costar, Corning, NY, USA) at a concentration of 5000 cells/well in 100 μL of DMEM and centrifuged for 10 min at 200× *g*. Methylcellulose was prepared as previously described [56]. Spheroids were formed for 3 days at 37 °C in a 5% CO_2_ humidified atmosphere.

### 4.3. COST Jet Plasma Setup

The COST plasma jet used in this work is described elsewhere [17,26,27]. The plasma was sustained at 250 VRMS and an operating frequency of 13.56 MHz. It was operated with a feed gas of He with 0%, 5% and 20% H_2_O vapour admixture achieved using the split He flow by passing part of it through an H_2_O-filled Drechsel flask. For the detailed setup description, see Supplementary Information (SI), Figure S6. H_2_O content is expressed as the percentage of the relative saturation of the feed gas at 21–22 °C, room temperature (RT) during the experiments.

### 4.4. Treatments

Before treatment, 3-day-old spheroids were washed once with PBS to remove the culture medium. The treatment regimen consisted of a single or two consecutive plasma treatments of 3 min each administered 24 h apart (referred to as 2× plasma treatment). Direct (spheroids in 200 µL of PBS in ULA plate) or indirect treatments (pPBS: 200 µL of PBS in ULA plate exposed to CAP, then transferred to spheroids) were performed with the different feed gas admixtures described above. Spheroids in 200 µL of untreated PBS were used as negative controls. A RONS mix solution of PBS containing the same concentrations of H_2_O_2_ and NO_2_^−^ detected in He + 20% H_2_O plasma-treated PBS was used to assess the role of the long- and short-lived species. Spheroids were incubated for 90 min with the corresponding treatment, after which it was replaced with warm, fresh medium prepared according to the downstream experiment.

### 4.5. Cytotoxicity Assay

The Cytotox Green reagent (10 nM, Essen Biosciences) was used for real-time quantification of cell death. Plates were incubated in the IncuCyte Live-Cell Analysis System (Sartorius, Ann Arbor, MI, USA) and images were collected every 24 h for 7 days. Data were analysed with the IncuCyte ZOOM version 2016B (Essen BioScience) to collect the following metrics: (1) spheroid core area, corresponding to the proliferative region only (red live cells; calculated as the mean fluorescent object area); (2) total spheroid area, corresponding to the combined viable, proliferative core and the Cytotox Green^+^ region (total spheroid region measured from phase contrast images); and (3) the amount of Cytotox Green^+^ in treated spheroids (confluence percentage of the image area occupied by green objects) normalized to the untreated control at each time point, representing the cytotoxic effect of the treatment.

### 4.6. Cell Migration Assay

A 2D migration assay was done in 96-well, flat-bottomed plates coated with 0.1% (*v*/*v*) gelatin [57]. Unbound gelatin was rinsed twice with PBS and wells were blocked with 1% (*w*/*v*) BSA in PBS for 1 h (RT). Spheroids were gently transferred to a migration plate containing 200 µL of DMEM supplemented with 2% FBS and 2 mM L-Glutamine. Spheroids were allowed to adhere to the plate for 30 min before imaging. Images were collected using the EVOS XL Core Cell Imaging System (Life Technologies). Migration was scored using ImageJ. To calculate the migration, the following formula was used: %Migration area = 100*(area T_x_-T_0_/area T_0_), where T_0_ corresponds to the migrated area at t = 0 h and T_x_ corresponds to the migrated area at each time point assessed.

### 4.7. Immunohistochemical Analysis

Spheroids were collected immediately after the second treatment and fixed with 4% paraformaldehyde for 24 h at 4 °C. Fixed spheroids were transferred to a 4% agarose mould as described before [58]. The agarose pads were paraffin-embedded. Sections of 5 µm were cut, deparaffinised and rehydrated prior to staining. Antigen retrieval was performed with citrate buffer (10 mM, pH 6), at 96 °C for 20 min. Sections were permeabilised in 0.1% Tween-20 and blocked with 3% H_2_O_2_ in PBS (10 min, RT). The primary antibody incubation was 40 min at RT (1/75 dilution; Mouse Anti-Human Ki-67 Antigen, Clone MIB-1, Agilent, Santa Clara, CA, USA), followed by incubation with the secondary antibody (30 min at RT; Envision Flex HRP). Diaminobenzidine was used to visualize positive staining and haematoxylin to counterstain. Sections were imaged with Zeiss AxioImager Z1 microscope (Carl Zeiss, Göttingen, Germany) equipped with an AxioCam MR ver.3.0. The number of ki67^+^ and haematoxylin^+^ cells was counted using ImageJ and the plugin IHC Profiler [59]. %ki67^+^ cells = (number ki67^+^ cells/number haematoxylin^+^ cells)*100.

### 4.8. Electron Paramagnetic Resonance (EPR) Spectroscopy

The presence of hydroxyl radical (•OH), ozone (O_3_)/atomic oxygen (O), singlet oxygen (O2(a1Δg)) and superoxide radical anions (O_2_•^−^) was assessed by EPR using spin trapping, as described elsewhere [14,31]. PBS solutions of spin traps 2,2,6,6-tetramethylpiperidine (TEMP, ≥99%, Sigma Aldrich, Overijse, Belgium), 5,5-dimethyl-1-pyrroline *N*-oxide (DMPO, ≥98%, Sigma Aldrich) and 5-(diethoxyphosphoryl)-5-methyl-1-pyrroline *N*-oxide (DEPMPO, ≥99%, Santa Cruz Biotechnology, Heidelberg, Germany) were freshly prepared at 0.1 M concentration prior to use. The O_2_(a^1^Δg) scavenger sodium azide (NaN_3_, ≥99%, Sigma Aldrich) at a 0.1 M concentration was added to a solution of TEMP to distinguish between the amount of 2,2,6,6-tertramethylpiperidine 1-oxyl (TEMPO) formed from O_3_/O and that from O_2_(a^1^Δg) [6,30]. In a typical experiment, 200 µL of PBS containing spin traps were exposed to plasma for 1–4 min.

The •NO radical was monitored using spin traps 2-phenyl-4,4,5,5-tetramethylimidazoline 1-oxyl 3-oxide (PTIO; ≥98%, Enzo Life Sciences, Antwerp, Belgium) and *N*-methyl-D-glucamine dithiocarbamate (MGD)-iron(II) complex (MGD)_2_Fe^2+^ [30,34,35]. In the first case, 0.2 mM solution of PTIO was exposed to plasma, after which it was immediately analysed by EPR to assess the formation of 2-phenyl-4,4,5,5-tetramethylimidazoline 1-oxyl (PTI). To prepare (MGD)_2_Fe^2+^, 100 µL of 4 mM solution of FeSO_4_•7H_2_O (≥99%, Sigma Aldrich, Overijse, Belgium) were mixed with 100 µL of 20 mM solution of MGD sodium monohydrate (≥98%, Enzo Life Sciences). To minimize the oxidation of Fe^2+^ to Fe^3+^ and the loss of the paramagnetic nature of the complex, both solutions were prepared in PBS that was degassed with Ar (99.998%, Praxair, Schoten, Belgium) for 2 min, and 50 µL of 20 mM solution of Na_2_S_2_O_3_ (99%, Sigma Aldrich) were added after the exposure [34,60].

EPR measurements were carried out on a Magnettech MiniScope MS 200 spectrometer with the following parameters: frequency 9.4 GHz, power 5 dBm (3.16 mW) or 15 dBm (31.6 mW), modulation frequency 100 kHz, modulation amplitude 0.1 mT, sweep time 40 s, time constant 0.1, sweep width 15 mT, number of scans 3. For the measurements, the analysed samples were contained in 50 μL glass capillaries (Hirschmann, Eberstadt, Germany). The concentrations reported were obtained via double integration (SpectrumViewer ver. 2.6.3 [61]) of the respective simulated spectra (NIH P.E.S.T. WinSIM software ver. 0.96 [62]) of the formed nitroxide radical adducts (Figure S2). For simulations, the hyperfine coupling constants were obtained from the available literature [63]. The calibration of the EPR signal (double integral intensity as a function of a radical concentration) was carried out using a stable radical 4-hydroxy-2,2,6,6-tetramethylpiperidine 1-oxyl (96%, Sigma Aldrich), as previously described [27].

### 4.9. Colourimetric Assays

Colourimetric assays were used to quantify H_2_O_2_, nitrite (NO_2_^−^) and nitrate (NO_3_^−^) in pPBS in the absence of spheroids. The potassium titanium (IV) oxalate method was used to measure H_2_O_2_ as described elsewhere [40,64]. Immediately after plasma treatment, 50 µL of 80 mM NaN_3_ solution, 200 µL of pPBS and 50 µL of 0.1 M K_2_TiO(C_2_O_4_)_2_•2H_2_O (≥98% Ti basis, Sigma Aldrich) were mixed and analyzed by UV-Vis absorption using a spectrophotometer (Thermo Fischer Genesys 6) at 400 nm. The concentrations of NO_2_^−^ and NO_3_^−^ were measured using the Nitrate/Nitrite Colourimetric Assay Kit (Cayman Chemical, Ann Arbor, Michigan, USA) [65]. For NO_2_^−^ detection, 100 µL of pPBS were mixed with 50 µL of Griess Reagent 1 and 50 µL of Griess Reagent 2, incubated for 6 min. Samples were measured in a 96 flat-bottom well plate using the BIO-RAD iMark microplate reader at 540 nm. For NO_3_^−^ detection, 80 µL of pPBS were mixed with 10 µL of nitrate reductase and 10 µL of nitrate reductase cofactor and incubated for 1h at RT. This way, all NO_3_^−^ is converted into NO_2_^−^, which was measured. The amount of NO_3_^−^ was calculated as the difference of the two values obtained.

The calibration was performed using solutions of H_2_O_2_ (30 wt%, Fisher Scientific S.P.R.L., Brussels, Belgium) and NaNO_2_ (provided in the kit). Background absorbance and PBS evaporation were accounted for in the final concentration values.

### 4.10. Statistical Analysis

One- (ki67 staining) and two-way ANOVA (cytotoxicity and migration assays) followed by Tukey’s multiple comparison test were performed using Prism v.6.01 (GraphPad Software, La Jolla, CA, USA). Statistical significance was set at *p* < 0.05.

## 5. Conclusions

We conclude that CAP treatment can effectively reduce 3D glioblastoma spheroid growth, cell migration and cell proliferation. Our results indicate the importance of the CAP-generated short-lived species for the growth inhibition and cell cytotoxicity of solid glioblastoma tumours, as they are necessary to achieve a sustained reduction of 3D glioblastoma spheroids in vitro. These results demonstrate the potential of CAP therapies for cancer treatment.

## Supplementary Materials

The following are available online at http://www.mdpi.com/2072-6694/10/11/394/s1, Figure S1: Cytotoxic effect of single plasma treatment in U87-Red and U251-Red spheroids; Figure S2: Calculation of the concentration of spin radical adduct present in the sample using DMPO; Figure S3: Representative experimental and simulated EPR spectra of the radical adducts of DEPMPO formed in pPBS; Figure S4: DMPO-OH adduct formation as a function of plasma treatment time; Figure S5: Representative experimental and simulated EPR spectra of TEMPO: impurities in an untreated PBS sample and TEMPO measured in pPBS; Figure S6: Schematic of the COST plasma jet.

## References

[1] de Vleeschouwer S. (2017). Glioblastoma [Internet].

[2] Koshy M., Villano J.L., Dolecek T.A., Howard A., Mahmood U., Chmura S.J., Weichselbaum R.R., McCarthy B.J. (2012). Improved survival time trends for glioblastoma using the SEER 17 population-based registries. J. Neurooncol..

[3] Ostrom Q.T., Gittleman H., Xu J., Kromer C., Wolinsky Y., Kruchko C., Barnholtz-Sloan J.S. (2016). CBTRUS statistical report: Primary brain and other central nervous system tumors diagnosed in the United States in 2009–2013. Neuro. Oncol..

[4] Yan D., Sherman J.H., Keidar M. (2017). Cold atmospheric plasma, a novel promising anti-cancer treatment modality. Oncotarget.

[5] Weltmann K.D., von Woedtke T. (2017). Plasma medicine—Current state of research and medical application. Plasma. Phys. Contr. F..

[6] Lu X., Naidis G.V., Laroussi M., Reuter S., Graves D.B., Ostrikov K. (2016). Reactive species in non-equilibrium atmospheric-pressure plasmas: Generation, transport, and biological effects. Phys. Rep..

[7] Graves D.B. (2014). Reactive species from cold atmospheric plasma: Implications for cancer therapy. Plasma Process. Polym..

[8] Furuta R., Kurake N., Ishikawa K., Takeda K., Hashizume H., Tanaka H., Kondo H., Sekine M., Hori M. (2017). Intracellular responses to reactive oxygen and nitrogen species, and lipid peroxidation in apoptotic cells cultivated in plasma-activated medium. Plasma Process. Polym..

[9] De Backer J., Razzokov J., Hammerschmid D., Mensch C., Hafideddine Z., Kumar N., van Raemdonck G., Yusupov M., van Doorslaer S., Johannessen C. (2018). The effect of reactive oxygen and nitrogen species on the structure of cytoglobin: A potential tumor suppressor. Redox Biol..

[10] Hirst A.M., Simms M.S., Mann V.M., Maitland N.J., O’Connell D., Frame F.M. (2015). Low-temperature plasma treatment induces DNA damage leading to necrotic cell death in primary prostate epithelial cells. Br. J. Cancer.

[11] Lin A.G., Xiang B., Merlino D.J., Baybutt T.R., Sahu J., Fridman A., Snook A.E., Miller V. (2018). Non-thermal plasma induces immunogenic cell death in vivo in murine CT26 colorectal tumors. OncoImmunology.

[12] Dezest M., Chavatte L., Bourdens M., Quinton D., Camus M., Garrigues L., Descargues P., Arbault S., Burlet-Schiltz O., Casteilla L. (2017). Mechanistic insights into the impact of cold atmospheric pressure plasma on human epithelial cell lines. Sci. Rep..

[13] Graves D.B. (2014). Low temperature plasma biomedicine: A tutorial review. Phys. Plasmas..

[14] Yan D.Y., Talbot A., Nourmohammadi N., Sherman J.H., Cheng X.Q., Keidar M. (2015). Toward understanding the selective anticancer capacity of cold atmospheric plasma-A model based on aquaporins. Biointerphases.

[15] Van der Paal J., Neyts E.C., Verlackt C.C.W., Bogaerts A. (2016). Effect of lipid peroxidation on membrane permeability of cancer and normal cells subjected to oxidative stress. Chem. Sci..

[16] Doskey C.M., Buranasudja V., Wagner B.A., Wilkes J.G., Du J., Cullen J.J., Buettner G.R. (2016). Tumor cells have decreased ability to metabolize H_2_O_2_: Implications for pharmacological ascorbate in cancer therapy. Redox Biol..

[17] Vermeylen S., De Waele J., Vanuytsel S., De Backer J., Van der Paal J., Ramakers M., Leyssens K., Marcq E., Van Audenaerde J., Smits E.L.J. (2016). Cold atmospheric plasma treatment of melanoma and glioblastoma cancer cells. Plasma Process. Polym..

[18] Koritzer J., Boxhammer V., Schafer A., Shimizu T., Klampfl T.G., Li Y.F., Welz C., Schwenk-Zieger S., Morfill G.E., Zimmermann J.L. (2013). Restoration of sensitivity in chemo-resistant glioma cells by cold atmospheric plasma. PLoS ONE.

[19] Tanaka H., Mizuno M., Ishikawa K., Takeda K., Kondo H., Sekine M., Hashizume H., Nakamura K., Kajiyama H., Okazaki Y. (2018). Similarities and differences in the cellular responses between plasma-activated medium-treated glioblastomas and plasma-activated Ringer’s lactate solution-treated glioblastomas. Clin. Plasma. Med..

[20] Kurake N., Tanaka H., Ishikawa K., Kondo T., Sekine M., Nakamura K., Kajiyama H., Kikkawa F., Mizuno M., Hori M. (2016). Cell survival of glioblastoma grown in medium containing hydrogen peroxide and/or nitrite, or in plasma-activated medium. Arch. Biochem. Biophys..

[21] Son B., Lee S., Youn H., Kim E., Kim W., Youn B. (2017). The role of tumor microenvironment in therapeutic resistance. Oncotarget.

[22] Judee F., Fongia C., Ducommun B., Yousfi M., Lobjois V., Merbahi N. (2016). Short and long time effects of low temperature Plasma Activated Media on 3D multicellular tumor spheroids. Sci. Rep..

[23] Merbahi N., Chauvin J., Vicendo P., Judee F. (2017). Effects of plasma activated medium on head and neck FaDu cancerous cells: Comparison of 3D and 2D response. Anticancer Agents Med. Chem..

[24] Plewa J.-M., Yousfi M., Frongia C., Eichwald O., Ducommun B., Merbahi N., Lobjois V. (2014). Low-temperature plasma-induced antiproliferative effects on multi-cellular tumor spheroids. New J. Phys..

[25] Chen Z., Simonyan H., Cheng X., Gjika E., Lin L., Canady J., Sherman J.H., Young C., Keidar M. (2017). A novel micro cold atmospheric plasma device for glioblastoma both in vitro and in vivo. Cancers.

[26] Golda J., Held J., Redeker B., Konkowski M., Beijer P., Sobota A., Kroesen G., Braithwaite N.S., Reuter S., Turner M. (2016). Concepts and characteristics of the ’COST reference microplasma jet’. J. Phys. D Appl. Phys..

[27] Gorbanev Y., Verlackt C.C.W., Tinck S., Tuenter E., Foubert K., Cos P., Bogaerts A. (2018). Combining experimental and modelling approaches to study the sources of reactive species induced in water by the COST RF plasma jet. Phys. Chem. Chem. Phys..

[28] Katt M.E., Placone A.L., Wong A.D., Xu Z.S., Searson P.C. (2016). In vitro tumor models: Advantages, disadvantages, variables, and selecting the right platform. Front. Bioeng. Biotechnol..

[29] Achilli T.M., Meyer J., Morgan J.R. (2012). Advances in the formation, use and understanding of multi-cellular spheroids. Expert Opin. Biol. Ther..

[30] Takamatsu T., Uehara K., Sasaki Y., Miyahara H., Matsumura Y., Iwasawa A., Ito N., Azuma T., Kohno M., Okino A. (2014). Investigation of reactive species using various gas plasmas. RSC Adv..

[31] Gorbanev Y., O’Connell D., Chechik V. (2016). Non-thermal plasma in contact with water: The origin of species. Chem. Eur. J..

[32] Gorbanev Y., Soriano R., O’Connell D., Chechik V. (2016). An atmospheric pressure plasma setup to investigate the reactive species formation. J. Vis. Exp..

[33] Elg D.T., Yang I.W., Graves D.B. (2017). Production of TEMPO by O atoms in atmospheric pressure non-thermal plasma–liquid interactions. J. Phys. D Appl. Phys..

[34] Gorbanev Y., Stehling N., O’Connell D., Chechik V. (2016). Reactions of nitroxide radicals in aqueous solutions exposed to non-thermal plasma: Limitations of spin trapping of the plasma induced species. Plasma. Sources Sci. Technol..

[35] Chauvin J., Judée F., Yousfi M., Vicendo P., Merbahi N. (2017). Analysis of reactive oxygen and nitrogen species generated in three liquid media by low temperature helium plasma jet. Sci. Rep..

[36] Kondeti V.S.S.K., Phan C.Q., Wende K., Jablonowski H., Gangal U., Granick J.L., Hunter R.C., Bruggeman P.J. (2018). Long-lived and short-lived reactive species produced by a cold atmospheric pressure plasma jet for the inactivation of *Pseudomonas aeruginosa* and *Staphylococcus aureus*. Free Radic. Biol. Med..

[37] Lukes P., Dolezalova E., Sisrova I., Clupek M. (2014). Aqueous-phase chemistry and bactericidal effects from an air discharge plasma in contact with water: Evidence for the formation of peroxynitrite through a pseudo-second-order post-discharge reaction of H_2_O_2_ and HNO_2_. Plasma Sources Sci. Technol..

[38] Wende K., Williams P., Dalluge J., Van Gaens W., Aboubakr H., Bischof J., von Woedtke T., Goyal S.M., Weltmann K.D., Bogaerts A. (2015). Identification of the biologically active liquid chemistry induced by a nonthermal atmospheric pressure plasma jet. Biointerphases.

[39] Ikawa S., Tani A., Nakashima Y., Kitano K. (2016). Physicochemical properties of bactericidal plasma-treated water. J. Phys. D Appl. Phys..

[40] Van Boxem W., Van der Paal J., Gorbanev Y., Vanuytsel S., Smits E., Dewilde S., Bogaerts A. (2017). Anti-cancer capacity of plasma-treated PBS: Effect of chemical composition on cancer cell cytotoxicity. Sci. Rep..

[41] Ravi M., Paramesh V., Kaviya S.R., Anuradha E., Solomon F.D.P. (2015). 3D Cell culture systems: Advantages and applications. J. Cell. Physiol..

[42] Yan D.Y., Xiao H.J., Zhu W., Nourmohammadi N., Zhang L.G., Bian K., Keidar M. (2017). The role of aquaporins in the anti-glioblastoma capacity of the cold plasma-stimulated medium. J. Phys. D Appl. Phys..

[43] Wang M., Holmes B., Cheng X., Zhu W., Keidar M., Zhang L.G. (2013). Cold atmospheric plasma for selectively ablating metastatic breast cancer cells. PLoS ONE.

[44] Volotskova O., Shashurin A., Stepp M.A., Pal-Ghosh S., Keidar M. (2011). Plasma-controlled cell migration: Localization of cold plasma-cell interaction region. Plasma Med..

[45] Volotskova O., Hawley T.S., Stepp M.A., Keidar M. (2012). Targeting the cancer cell cycle by cold atmospheric plasma. Sci. Rep..

[46] Salazar-Ramiro A., Ramirez-Ortega D., de la Cruz V.P., Hernandez-Pedro N.Y., Gonzalez-Esquivel D.F., Sotelo J., Pineda B. (2016). Role of redox status in development of glioblastoma. Front. Immunol..

[47] Naciri M., Dowling D., Al-Rubeai M. (2014). Differential sensitivity of mammalian cell lines to non-thermal atmospheric plasma. Plasma Process Polym..

[48] Otto T., Sicinski P. (2017). Cell cycle proteins as promising targets in cancer therapy. Nat. Rev. Cancer.

[49] Jenkins N.C., Liu T., Cassidy P., Leachman S.A., Boucher K.M., Goodson A.G., Samadashwily G., Grossman D. (2011). The p16(INK4A) tumor suppressor regulates cellular oxidative stress. Oncogene.

[50] Sablina A.A., Budanov A.V., Ilyinskaya G.V., Agapova L.S., Kravchenko J.E., Chumakov P.M. (2005). The antioxidant function of the p53 tumor suppressor. Nat. Med..

[51] Ma Y., Ha C.S., Hwang S.W., Lee H.J., Kim G.C., Lee K.-W., Song K. (2014). Non-thermal atmospheric pressure plasma preferentially induces apoptosis in p53-mutated cancer cells by activating ROS stress-response pathways. PLoS ONE.

[52] Guerrero-Preston R., Ogawa T., Uemura M., Shumulinsky G., Valle B.L., Pirini F., Ravi R., Sidransky D., Keidar M., Trink B. (2014). Cold atmospheric plasma treatment selectively targets head and neck squamous cell carcinoma cells. Int. J. Mol. Med..

[53] Li H., Lei B., Xiang W., Wang H., Feng W., Liu Y., Qi S. (2017). Differences in protein expression between the U251 and U87 cell lines. Turk. Neurosurg..

[54] Yang C., Zhang J., Ding M., Xu K., Li L., Mao L., Zheng J. (2018). Ki67 targeted strategies for cancer therapy. Clin. Transl. Oncol..

[55] Liedtke K.R., Bekeschus S., Kaeding A., Hackbarth C., Kuehn J.-P., Heidecke C.-D., von Bernstorff W., von Woedtke T., Partecke L.I. (2017). Non-thermal plasma-treated solution demonstrates antitumor activity against pancreatic cancer cells in vitro and in vivo. Sci. Rep..

[56] Longati P., Jia X.H., Eimer J., Wagman A., Witt M.R., Rehnmark S., Verbeke C., Toftgard R., Lohr M., Heuchel R.L. (2013). 3D pancreatic carcinoma spheroids induce a matrix-rich, chemoresistant phenotype offering a better model for drug testing. BMC Cancer.

[57] Vinci M., Box C., Zimmermann M., Eccles S.A., Moll J., Colombo R. (2013). Tumor spheroid-based migration assays for evaluation of therapeutic agents. Target identification and Validation in Drug Discovery: Methods and Protocols.

[58] Ivanov D.P., Grabowska A.M. (2017). Spheroid arrays for high-throughput single-cell analysis of spatial patterns and biomarker expression in 3D. Sci. Rep..

[59] Varghese F., Bukhari A.B., Malhotra R., De A. (2014). IHC Profiler: An open source plugin for the quantitative evaluation and automated scoring of immunohistochemistry images of human tissue samples. PLoS ONE.

[60] Nedeianu S., Pali T. (2002). EPR spectroscopy of common nitric oxide–spin trap complexes. Cell Mol. Biol. Lett..

[61] Timmerman E. Spectrum Viewer 2.6.3. http://www.phys.tue.nl/people/etimmerman/specview/.

[62] Duling D.R. (1994). Simulation of Multiple Isotropic Spin Trap EPR Spectra. J Magn Reson B..

[63] Database, N.I.S.T. http://tools.niehs.nih.gov/stdb/index.cfm.

[64] Privat-Maldonado A., Gorbanev Y., O’Connell D., Vann R., Chechik V., Woude M.W.v.d. (2017). Non-target biomolecules alter macromolecular changes induced by bactericidal low-temperature plasma. IEEE Trans. Radiat. Plasma Med. Sci..

[65] Gibson A.R., McCarthy H.O., Ali A.A., O’Connell D., Graham W.G. (2014). Interactions of a non-thermal atmospheric pressure plasma effluent with PC-3 prostate cancer cells. Plasma Process Polym..

