# Supplementary material for: Reduction of Human Glioblastoma Spheroids Using Cold Atmospheric Plasma: The Combined Effect of Short- and Long-Lived Reactive Species
Source: Cancers (Basel). 2018 Oct 23;10(11):394. doi: 10.3390/cancers10110394 (PMC6266784; doi:10.3390/cancers10110394)
Supplement: Supplementary file 1 [file cancers-10-00394-s001.pdf]

Supplementary material

Reduction of Human Glioblastoma Spheroids Using Cold Atmospheric Plasma: The Combined Effect of Short- and Long-Lived Reactive Species

Angela Privat-Maldonado, Yury Gorbanev, Sylvia Dewilde, Evelien Smits and Annemie Bogaerts

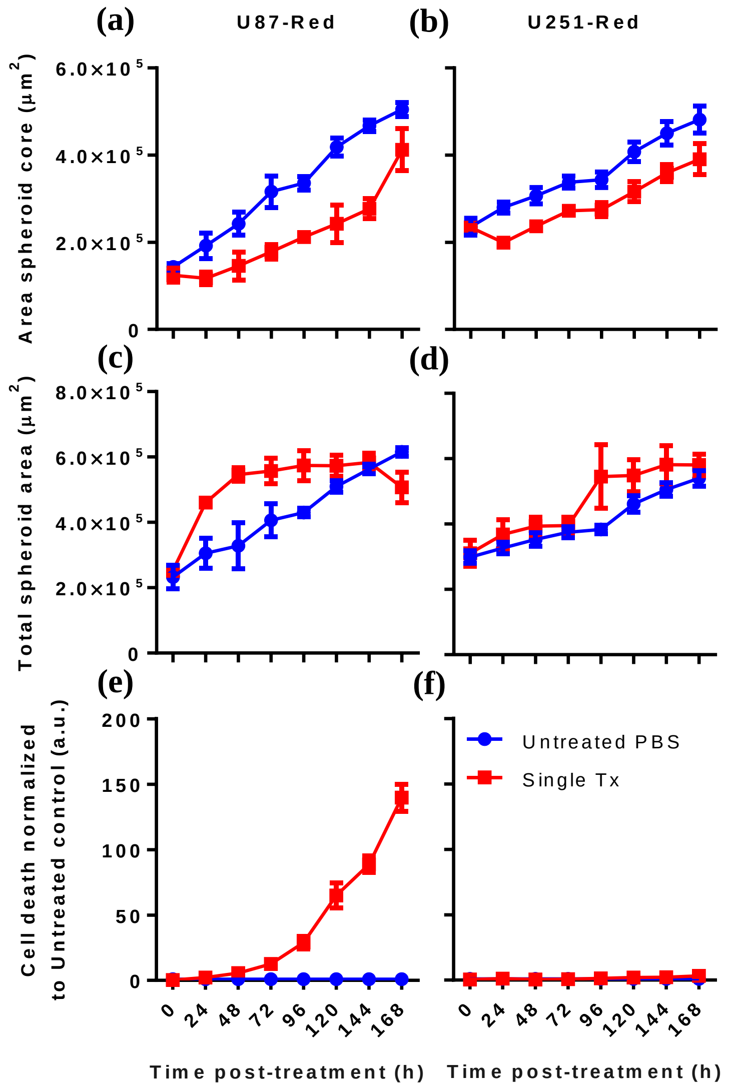

Figure S1. Cytotoxic effect of single plasma treatment in U87-Red and U251-Red spheroids. Spheroids were exposed to untreated PBS or a single direct plasma treatment (3 min, He + 20% H_2O; single Tx). Plasma-treated spheroids (**a**,**b**) presented a delayed growth, but it was not inhibited. (**c**,**d**) Similar total spheroid areas were observed between the untreated and plasma-treated spheroids 7 days post-treatment. (**e**,**f**) The treatment was cytotoxic for U87-Red, but not for U251-Red. Data representative of 2 independent experiments, 4–6 spheroids per condition. Mean ± SD. Tx = Treatment.

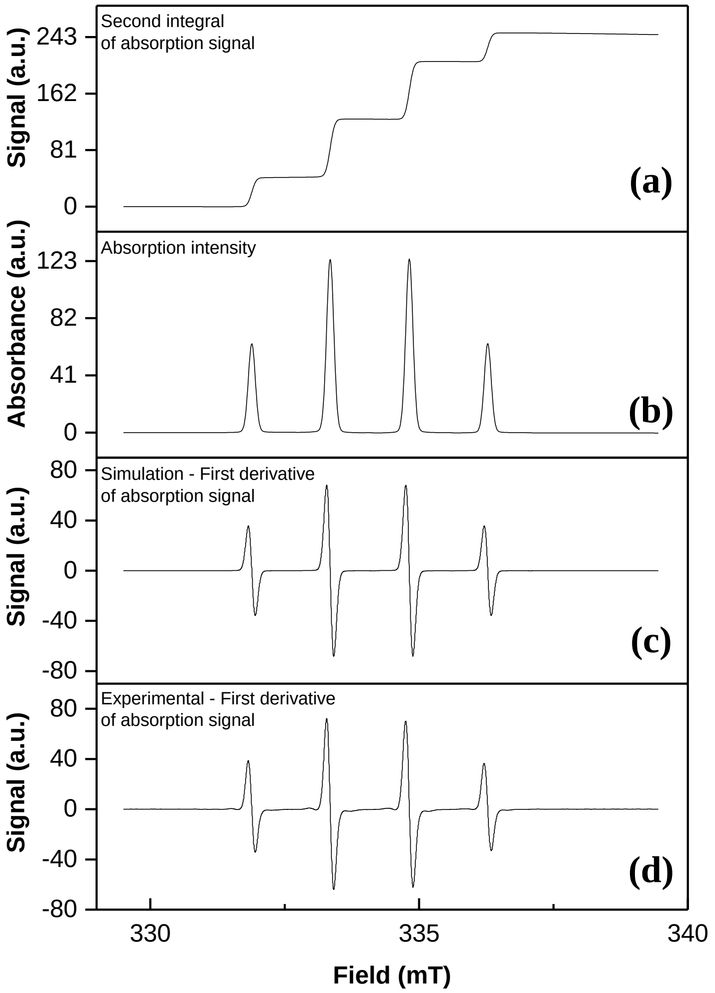

Figure S2. Calculation of the concentration of spin radical adduct present in the sample using DMPO. (**a**) Area under the absorption signal, second integral of the absorption signal. The value obtained is proportional to the number of radicals present in the sample. (**b**) Absorption signal, obtained through integration of the first derivative. (**c**) First derivative of the absorption signal, simulated spectrum. (**d**) First derivative of the absorption signal, experimentally obtained spectrum.

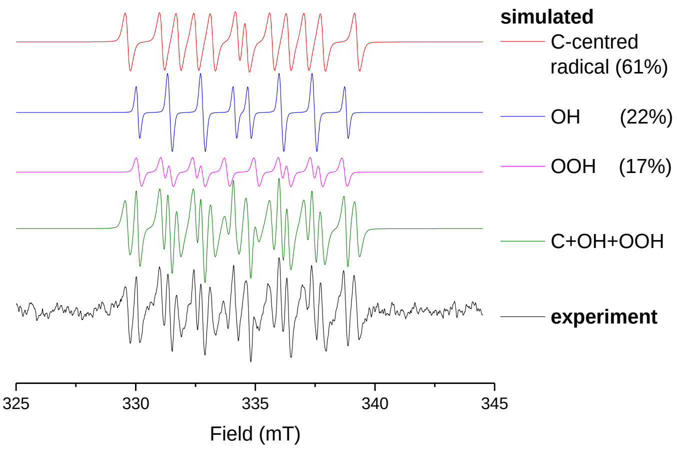

Figure S3. Representative experimental and simulated EPR spectra of the radical adducts of DEPMPO formed in pPBS. Radical adduct hyperfine values (mT): DEPMPO-OOH aN = 1.34, aH = 0.99, aP = 4.92; DEPMPO-OH aN = 1.38, aH = 1.31, aP = 4.67; DEPMPO-C aN = 1.43, aH = 2.13, aP = 4.59. The contribution of each adduct to the combined simulated spectrum is shown in %. Three adducts were detected: with O_2•⁻ /•OOH, •OH and C-centred radical [1]. The latter is formed via partial degradation of DEPMPO upon plasma treatment. This confirms that oxygen species are virtually absent in pPBS, as they will rapidly decay the C-radical [1,2]. It is worth noting that the O_2•⁻ (superoxide) and •OOH (hydroperoxyl) radicals are generally indistinguishable using nitrone spin traps (NST). Based on the pKa value, at physiological pH, O_2•⁻ exists mainly in the non-protonated form. However, the O_2•⁻ adduct of a spin trap gets protonated to form NST-OOH (and not NST- O_2•⁻) [3].

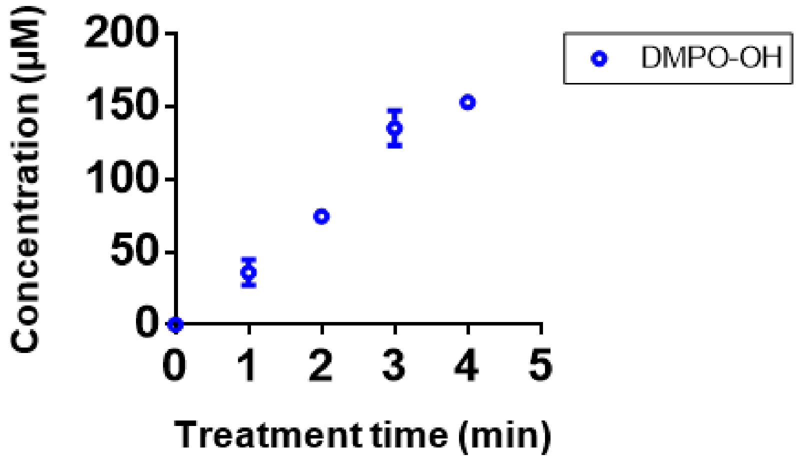

Figure S4. DMPO-OH adduct formation as a function of plasma treatment time. Plasma generated using He + 20% H_2O vapour saturation. Mean ± SD.

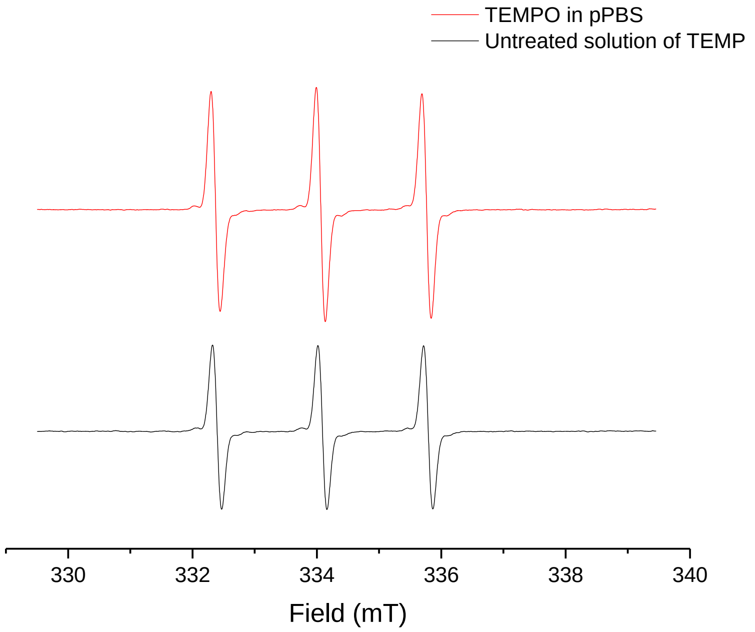

Figure S5. Representative experimental and simulated EPR spectra of TEMPO: impurities in an untreated PBS sample and TEMPO measured in pPBS. The signal observed in the untreated sample results from impurities. The difference was constant in all three conditions (below 9 μM), suggesting that these ROS ($O/O_3/O_2(a_1\Delta g)$) were not the main species causing the observed biological effect. Hyperfine value: a_N = 1.70 mT.

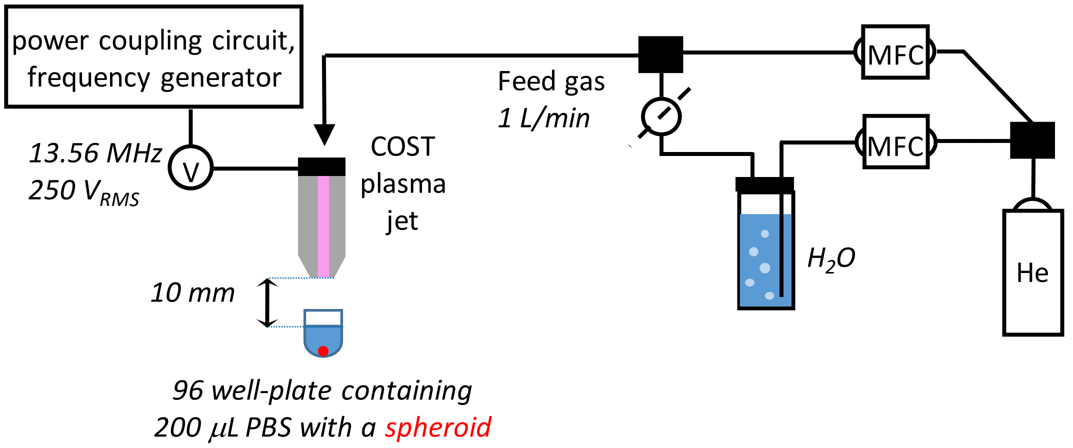

Figure S6. Schematic of the COST plasma jet. The plasma jet was operated with He gas split into two lines and it was controlled by mass flow controllers (MFC). Part of He flow (50 or 200 mL/min) was passed through an H_2O-filled Drechsel flask, to achieve relative H_2O saturation in the feed gas of 5% or 20%, respectively. The total feed gas flow was 1 L/min in all cases. With this method, the H_2O vapour content was varied during experiments (H_2O vapour content expressed as the percentage of the relative saturation of the feed gas at RT during the experiments). The plasma was sustained at 250 V_{RMS} and at an operating frequency of 13.56 MHz. The distance between the plasma jet nozzle and the surface of PBS in 96-well ULA plates was set at 10 mm. For direct treatments, glioblastoma spheroids in 200 μL of PBS were exposed to plasma. For indirect treatments (pPBS), only 200 μL of PBS were plasma-treated in the absence of spheroids.

References

1. Gorbanev, Y.; O'Connell, D.; Chechik, V. Non-thermal plasma in contact with water: the origin of species. *Chem. Eur. J.* **2016**, *22*, 3496–3505, doi:10.1002/chem.201503771.
2. Maillard, B.; Ingold, K.U.; Scaiano, J.C. Rate constants for the reactions of free radicals with oxygen in solution. *J. Am. Chem. Soc.* **1983**, *105*, 5095–5099, doi:10.1021/ja00353a039.
3. Burgett, R.A.; Bao, X.; Villamena, F.A. Superoxide radical anion adduct of 5,5-dimethyl-1-pyrroline n-oxide (DMPO). 3. Effect of mildly acidic ph on the thermodynamics and kinetics of adduct formation. *J. Phys. Chem. A*, **2008**, *112*, 2447–2455, doi:10.1021/jp7107158.

© 2018 by the authors. Licensee MDPI, Basel, Switzerland. This article is an open access article distributed under the terms and conditions of the Creative Commons Attribution (CC BY) license (http://creativecommons.org/licenses/by/4.0/).
